# Bridging the legitimacy gap—translating theory into practical signposts for legitimate flood risk governance

**DOI:** 10.1007/s10113-017-1195-4

**Published:** 2017-07-21

**Authors:** Meghan Alexander, Neelke Doorn, Sally Priest

**Affiliations:** 1 Sustainability Research Institute, School of Earth & Environment, University of Leeds, Leeds, LS2 9JT UK; 2 Department of Technology, Policy and Management, Delft University of Technology, P.O. Box 5015, 2628 BX Delft, The Netherlands; 3 Flood Hazard Research Centre, Middlesex University, The Burroughs, Hendon, London, NW4 4BT UK

**Keywords:** Flood risk governance, Legitimacy, Justice, Equity, Participation, Representative deliberation

## Abstract

Legitimacy is widely regarded as a founding principle of ‘good’ and effective governance, yet despite intense academic debate and policy discourse, the concept remains conceptually confusing and poorly articulated in practice. To bridge this gap, this research performed an interpretive thematic analysis of academic scholarship across public administration, public policy, law, political science, and geography. Four core themes were identified in relation to *representative deliberation*, *procedural and distributive equity and justice*, and *socio-political acceptability*, with numerous sub-themes therein. In an attempt to clarify conceptual confusion, this paper grounds these theoretical debates in the context of flood risk governance where numerous legitimacy dilemmas exist. A number of questions are presented as conceptual ‘signposts’ to encourage reflexive governance in the future. Thus, more broadly, we assert the importance of bringing legitimacy to the forefront of contemporary flood risk governance discourse and practice, moving beyond the realm of academic reflection.

## Introduction

Legitimacy has been the subject of growing attention in contemporary debates of climate change adaptation (Paavola and Adger 2006; Cashmore and Wejs 2014), earth system governance (Biermann and Gupta 2011), and, to a lesser extent, flood risk governance (Alexander et al. 2016a; Mees et al. 2017). In the context of future uncertainty and projected escalations of flood risk (Hirabayashi et al. 2013), legitimate governance is seen as a cornerstone for effective risk management and adaptation, as well as steering action at the local scale (Termeer et al. 2011; Cosens 2013). In order to address inherently uncertain and complex problems such as flooding, the diversification of risk management strategies is seen as an essential for societal resilience (Driessen et al. 2016), yet this also brings to the fore new challenges for legitimate governance. The shift towards risk management away from traditional paradigms of defence raises questions about the distribution of responsibilities across a more diversified spectrum of public-private actors, how to decide where and how risk management strategies will be applied, and share the distribution of costs and benefits (e.g. Mees et al. 2014; van Buuren et al. 2014).

Although legitimacy is commonly regarded as a founding principle of ‘good governance’ (e.g. European Commission, 2001; OECD, 2015), governance is conceived as a threat to traditional notions of democratic legitimacy (e.g. Sørensen and Torfing 2005). Governance signifies the transition from centralised, state-led decision-making towards multi-layered forms of interaction, either across nested jurisdictional levels or through polycentric non-hierarchical formations (Marks and Hooghe 2004). Governance also signifies the inclusion of a broader range of actors (e.g. public, private, and civil society) and potential for various modes of governance in the realisation of collective goals (Driessen et al. 2012). With the State no longer necessarily playing a pivotal role in decision-making, it is becoming increasingly accepted that traditional constructs of legitimacy rooted in democratic theory must evolve (Papadopoulos 2000; Sørensen 2010; Dellas 2011).

This has spawned considerable academic debate concerning the hallmarks of legitimate governance. In the field of flood risk governance—defined by Alexander et al. (2016a: 39) as the actor networks, rules, resources, discourses, and multi-level coordination mechanisms through which flood risk management (FRM) is pursued—recent efforts have been made to transform conceptual discussions of legitimacy into frameworks for empirical assessment (Mees et al. 2014; Alexander et al. 2016a; Mees et al. 2017). Whilst these frameworks provide valuable academic tools for evaluating the legitimacy of governance arrangements, these remain situated amongst contested knowledge about what constitutes legitimacy. This is further complicated by the tendency for authors to employ the term without explicit definition and assume mutual understanding; however, as this review will highlight, this is not the case. Broadly speaking, democratic and political legitimacy typically occupy debates in political science (Scharpf 2000; Klijn and Skelcher 2007), alongside moral reasoning (Risse 2006; Jagers and Duus-Otterström 2008; Adger et al. 2017). Legal scholars have examined the implications of governance and legitimacy in the context of (shifting) legal principles, alongside matters of responsibility, accountability, procedural and substantive fairness, and the rule of law (e.g. Weber 1976; Ebbesson 2010; Termeer et al. 2011; Spagnuolo 2011; Driessen and van Rijswick 2011; Buijze 2013). Building upon these issues, public policy and administration scholarship seems to extend the view on legitimacy towards wider matters of public participation, social equity, and distributive justice (Few et al. 2007; Birnbaum 2016).

These concerns reflect the different emphases placed on the input, process (or ‘throughput’), and output legitimacy (Scharpf 1999; van Kersbergen and van Waarden 2004; Schmidt 2013). However, this arguably presents an overly simplified representation and portrays a linear progression towards a final product or end goal (i.e. output legitimacy), despite research emphasising the ongoing construction of legitimacy and possibility for input legitimacy without output legitimacy, or vice versa (Lindgren and Persson 2010; Mees et al. 2014). Furthermore, perceptions of legitimacy are neither static nor universal, but rather constructed through normative, socio-cultural frames, agendas, and interests, and thus variable from place to place and across various groups in society (Scharpf 2000; Johansson 2012; Bernstein 2011). As raised by Biermann and Gupta (2011: 1858), ‘a critical question becomes legitimacy in the eyes of whom?’

Whilst a ‘one size fits all’ conceptualization is clearly inappropriate, if legitimacy is to truly become embedded in the delivery, assessment, and monitoring of governance, there is a need to clarify and identify ‘signposts’ to assist those negotiating such endeavours. We hereby echo recent calls requesting that theoretical debates be translated into governance arrangements (e.g. Termeer et al. 2011). Drawing from a thematic analysis of academic literature, this research discerns several prominent themes, which are reviewed in turn. A range of illustrative examples are employed to demonstrate the complex space through which legitimacy discourses manifest in flood risk governance and are constructed through socio-cultural settings. In an effort to bridge the ‘legitimacy gap’ between academia and practice, a number of critical questions are put forward to guide policy-makers and practitioners in this field (although we also expect a degree of transferability to other aspects of environmental governance). Given the highly contextualised nature of legitimacy, rather than proposing indicators and benchmarks for success (e.g. Mees et al. 2014), these ‘signposts’ adopt an alternative stance that emphasises the importance of openly reflexive flood risk governance.

## Methodology: analysing academic constructions of legitimacy

In order to examine and synthesise academic constructions of legitimacy, this research performed an interpretive analysis of peer-reviewed articles. As the most comprehensive of the Abstract and Index databases, Scopus was used as a starting point for sampling. This was approached inductively through a Boolean and truncation search (legitimacy* AND governance), searching the title, abstract, and key words. Further limitations were then applied to the search, focusing on articles published or in press in peer-reviewed journals (as the source type) and published in English. Exclusions were made for non-relevant subject areas (e.g. medicine, engineering, computer science, biochemistry, mathematics), then limited to social and environmental sciences, producing 1565 articles.

Preliminary analysis of these results revealed the dominance of publications in the UK and USA, and an increasing trend in publications from the mid-1990s to today, with a significant rise in publications from 2005 onwards. Citation information and author-listed key words were exported as a csv file (referred to as the ‘mother sample’). Results were then filtered according to articles where ‘legitimacy’ formed an author-listed key word, amounting to 302 articles,^1^ originating across multiple fields of scholarship, including public administration, public policy, political science, law, and geography. Whilst these inclusion/exclusion criteria provided a necessary starting point for sampling the literature in a pragmatic way, we also employed a snowballing technique to identify additional literature cited within the sample. In particular, we focused on articles relevant for elaborating on key themes and purposively added these to the analysis. An additional ca. 50 articles were captured within the mother sample and via snowballing techniques.

**Fig. 1:**
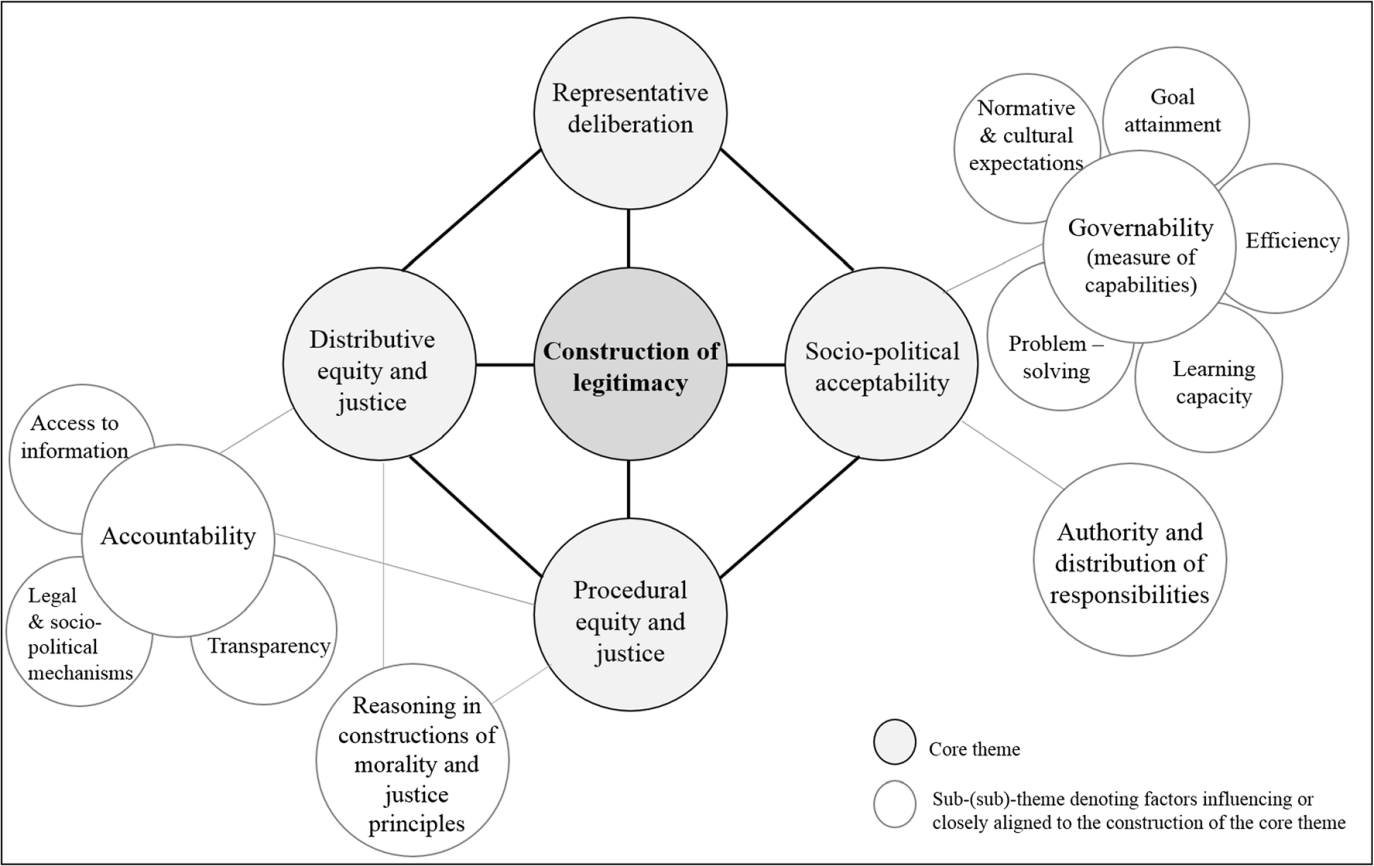
Thematically mapping constructions of legitimacy

Each article was downloaded into the qualitative analysis software package, NVivo, and subject to thematic analysis to unpick how legitimacy is theoretically framed within certain contexts and related to debates in governance. Themes were identified through an iterative and comparative process, coding for nuances and relationships between themes (Charmaz 2006). Central themes and sub-themes are outlined in Table 1 and illustrated in the thematic map presented in Fig. 1. As it would be confusing to draw all points of connectivity, we have intentionally organised the core themes in cyclic form to illustrate their interaction, whilst simultaneously portraying the ongoing process through which legitimacy is constructed or potentially deconstructed.

**Table 1:** Core themes in academic constructions of legitimacy

Theme	Explanation	Example articles
Representative deliberation	We coin the expression ‘representative deliberation’, drawing from contemporary debates in democratic theory and governance (sometimes referred to as interactive or network governance). This theme concerns the representation of stakeholders in participatory processes and the nature of deliberation. *Sub-themes* included the distribution of power and valuations of knowledge.	Klijn and Skelcher 2007; Few et al. 2007; Sørensen 2010; Dombrowski 2010; Dellas 2011; Häikiö 2012; Barnaud and Van Paassen 2013; Cheyne 2015; Mees et al. 2016a, 2017
Equity and justice	Theme relates to discussions of equity and justice in governance. The distinction is made between procedural elements (strongly linked to the theme on representative deliberation) and distributive debates (e.g. burden sharing). These debates are influenced by underlying justice principles and moral reasoning. Moreover, accountability is identified as an essential pre-requisite (with further requirements for transparency, access to information, as well as legal and socio-political mechanisms).	Paavola and Adger 2006; Termeer et al. 2011; Biermann and Gupta 2011; Gross-Camp et al. 2012; Penning-Rowsell and Priest 2015; Kaufmann et al. 2016; Hartmann and Spit 2016 Thaler and Hartmann 2016; Adger et al. 2017
Socio-political acceptability	This theme unpicks the various ways through which socio-political acceptability of governance and resulting outcomes/outputs are judged. This is sometimes referred to as ‘output legitimacy’ (e.g. Scharpf 1999). Two core sub-themes are identified. Firstly, *governability* indicates a measure of performance. Governance outcomes are accepted and legitimised for multiple reasons, including problem-solving capabilities, goal attainment, efficiency, and learning capacity, as well as normative and cultural expectations. Secondly, legitimation occurs through the acceptance of *authority and distribution of responsibilities*.	van Kersbergen and van Waarden 2004; Esty 2006; Biermann and Gupta 2011; Cashmore and Wejs 2014; Mees et al. 2014; van Buuren et al. 2014; Eriksen et al. 2015; Birnbaum 2016

In the forthcoming sections, we critically reflect on each theme in turn and elaborate on how these manifest in the context of flood risk governance, drawing from real-world examples to provide tangible reference points to ground the theoretical discussion. Legitimacy debates pivot around multiple types of actors, such as the state (government, elected officials, and public authorities), private citizens (individuals and householders, referred to as ‘the public’), voluntary organisations, non-governmental organisations (NGOs), and conservation groups, as well as market-based actors (e.g. insurers, small businesses). Moreover, ‘the public’ are not a homogenous group, but include those at risk of flooding, tax payers, and riparian land and property owners. The different values, interests, and agendas propagated by these groups invariably influence perspectives on legitimacy; therefore, this article employs a range of examples to demonstrate this. We do not presume that one perspective is more valuable over another, or impose norms for universally assessing legitimacy, as these may also vary across socio-cultural-normative settings. Our primary goal is to promote stronger engagement with the principles of legitimacy in flood risk governance and propose pragmatic signposts for translating the current academic debate into practice. Therefore, each section concludes with a series of critical questions to act as conceptual ‘signposts’ in guiding future reflexivity on legitimate flood risk governance. Although it is not possible to reference all articles reviewed, the following selections draw from those that best capture the range in the academic discourse on legitimacy.

## Representative deliberation

Legitimacy is widely framed in the context of democratic legitimacy (van Kersbergen and van Waarden 2004; Pierre and Peters 2005; Sørensen 2010) and multi-stakeholder participation (Few et al. 2007; Häikiö 2012; Mees et al. 2017). Observations have been made about the shifting relationship between the state and non-state actors (such as private citizens, businesses, NGOs), from once unidirectional, top-down diffusion of knowledge towards more multidirectional forms of knowledge exchange and participatory governance (e.g. Birnbaum 2016). This transition presents both a threat and opportunity for addressing legitimacy deficits.

Equality and deliberation are central to the normative foundations of democracy (Sørensen & Torfing 2005; Bernstein 2011). Whilst democratic equality asserts that those affected by a decision ‘have an equal access to influencing that decision’ (Sørensen 2010: 4), determining the boundaries of inclusion/exclusion are much debated. Traditional inclusion criteria based on national citizenship and territory have been called into question, with *levels of affectedness* now also deemed relevant. This allows for ‘tailor-made patterns of democratic inclusion’ (Sørensen 2010: 5). With this, observations have been made about the growing trend towards public and multi-stakeholder participation across a range of decision-making contexts (e.g. Berghofer et al. 2008; Dombrowski 2010; Häikiö 2012; Cheyne 2015; Johansson 2012, 2016).

However, it should be borne in mind that participation is motivated by different underlying rationales, which may not directly relate to the legitimation of governance. For instance, Mees et al. (2016a) show how efforts to ‘coproduce’ flood risk governance between public authorities and private citizens in selected European countries, may be driven by efforts to facilitate the transfer of risk responsibilities onto at-risk householders and propagate societal acceptance of alternative measures to flood defence. Birnbaum (2016) argues that public participation in the context of sustainable development planning primarily appears to have established as a professionally mediated exercise, seeking consensus as opposed to confrontation and serving conventional governing practices. In agreement, Few et al. (2007) note how participation is often used as a rhetoric for consultation, without any real redistribution of power. Therefore, in order to appropriately judge participatory quality, it is essential that such underlying motives are explicated.

Multi-stakeholder participation is not in itself a guarantee of legitimacy, but can both undermine as well as support pursuits of legitimate governance (Thaler and Hartmann 2016). Moreover, Mees et al. (2014) show how traditional forms of hierarchical governance (whereby interests are indirectly represented by elected officials acting for the common good) and emerging governance networks (i.e. potentially serving multiple interests) can both be perceived as legitimate. Nonetheless, as a means of improving ‘input legitimacy’, there is a strong consensus that participation ensures that different perspectives, values, and agendas of different stakeholders are represented and deliberated within decision-making (e.g. Dombrowski 2010).

The nature of deliberation is also pertinent. This necessitates forums for facilitating dialogue and negotiating potentially conflicting interests in the pursuit of collective action (Termeer et al. 2011). According to Sørensen (2010), interactive governance is particularly amenable to this democratic norm, providing that the right to dissimilar opinions is upheld. In turn, it is argued that deliberation across multiple stakeholders provides a pathway for increasing the quality of the output and, thus, outcome legitimacy (Scharpf 2000). However, this is somewhat dependent on the extent to which various perspectives are deliberated and weighted within the governance process. The latter requires a balancing of power; however, numerous research have documented how dominant voices and power elites may skew the representation of interests and advantage certain groups over others (Bernstein 2011; van Buuren et al. 2014).

On this front, Few et al. (2007) assert the importance of *avoiding the illusion of inclusion* and honestly communicating the instrumental goals of public participation. In the context of the UK coastal management, the authors observe the ‘containment of participation’ particularly where radical interventions are proposed (i.e. phased relocation). For anticipatory adaptation and complex environmental problems, more limited forms of engagement may be required; however, there needs to be some form of expectation management and clear delineation of participatory goals to avoid dissatisfaction and cultivate trust between governing authorities and the public.

In order to manage deliberation processes, the role of the ‘designers’ (or facilitator) of participatory processes as a neutral bystander or as a mediator for power asymmetries should also be considered. Barnaud and Van Paassen (2013: 21) propose a ‘critical companion’ posture, ‘whereby designers make explicit their assumptions and objectives regarding the social context so that local stakeholders can choose to accept them as legitimate or to reject them’. The ‘make-up’ of the participatory group should also be transparent. Indeed, there may be instances where public participation can be justifiably limited, such as situations where certified expertise is best placed to determine actions (Renn 2006; Hartmann and Spit 2016).

Despite the fact that participation is a specified objective in environmental policy and law (e.g. Water Framework Directive 2000/60/EC), in practice, this is delivered to varying degrees. Performing a cross-country comparison, Priest et al. (2016) examine the implementation of the EU Floods Directive (2007/60/EC) in England, France, Poland, Sweden, and the Netherlands. The authors adopt the stance that *effective public participation and access to justice* is necessary for legitimate goal attainment and, in turn, flood resilience. Although the Directive requests the involvement of interested parties in the production, review, and updating of FRM plans, specific details on the nature of participation are absent, thus leaving considerable scope for variation. In Poland, EU accession in 2004 is seen as a pivotal factor for change in flood risk governance and public participation (albeit consultative) has grown accordingly (Matczak et al. 2017). In contrast, this has had very little impact in England where more active (as opposed to passive) participatory initiatives have long been established (Priest et al. 2016). However, caution should be exercised in the interpretation of such findings or assuming that flood risk governance in one country is more or less legitimate than another. As this review will continue to demonstrate, constructions of legitimacy are indeed multi-faceted and contextually rooted.

Moving the debate forward, challenges remain about how to normalise and institutionalise legitimacy in the context of governance. To this, we were inspired by the seminal work of Sørensen (2010). Drawing from different epistemological standpoints within neo-institutional theory, whereby institutions are conceived as both shaping and being shaped by governance, Sørensen argues that informal institutional features (e.g. logics of appropriateness, normative codes, incentive structures) can support the establishment of *interactive democracy*. For instance, normative codes of conduct could encourage those participating in governance to legitimise their position by stating their representativeness to those affected and provide transparent accounts of their activities within this process to support democratic accountability. This might be further reinforced by incentive mechanisms which grant or withhold rights to participation. Logics of appropriateness (March and Olsen 2008) could also promote the importance of ‘input legitimacy’ and establish the governance arena as a place of deliberative democracy whereby all forms of knowledge and reasoning are valid. These suggestions could also hold merit for flood risk governance.

Combining these debates, we coin the expression ‘representative deliberation’. In order to minimise deficits in legitimacy on this count, we contend that critical reflexivity can be articulated through the following questions:

- What are the driving motivations and instrumental goals of stakeholder participation? (e.g. pursuit of knowledge, co-production, societal acceptance of pre-determined decisions?)
- What factors have determined access to participation in flood risk governance (i.e. inclusion/exclusion criteria)? How are these justified against instrumental goals?
- How are different perspectives represented and weighted within deliberation processes? What is the underlying justification for this?
- To what extent can participants make claims of representativeness?
- To what extent does the governance process foster democratic deliberation, where all views/knowledge types are respected and negotiated in the pursuit of a common goal? In what forum does this take place (e.g. talk-based interaction or some other form)?
- What is the role of the facilitator(s) in participatory processes? For example, do they adopt a ‘neutral’ stance or take deliberate intervention in group dynamics?
- Are participants of the governance process required to provide narrative accounts and/or document internal discussions to make the deliberation process transparent and accountable?

## Equity and justice in the construction of legitimacy

A second core theme discernible from this analysis pertains to equity and justice (Table 1). Social equity is concerned with qualities of fairness and is conceptually distinct from discussions of equality, despite often being discussed in tangent (Rawls 2001; Doorn 2015). Judgements of fairness are therefore tied to different principles of justice, including utilitarian, libertarian, egalitarian, pluralists, and Rawlsians (see Miller 1999). Several authors have demonstrated how these justice principles can manifest in different FRM approaches (Johnson et al. 2007; Thaler and Hartmann 2016); thus, what is deemed to be equitable (and thus legitimate) will vary across different socio-cultural, normative settings.

These debates are present in both procedural and distributive elements of governance, which are now reviewed in turn. However, it is also noteworthy that a considerable number of articles conduct these discussions without explicit reference to legitimacy. For example, in the flood context, considerations about the fair and just distribution of flood risk, and costs and benefits associated with FRM, are widespread (Fielding 2012; Walker and Burningham 2011; Chakraborty et al. 2014; Doorn 2015; Keessen et al. 2016; Thaler and Hartmann 2016). Alternatively, equity is sometimes framed as a distinct but allied concept to legitimacy (e.g. Adger et al. 2005). These articles were drawn upon as part of purposive sampling to further elaborate and illustrate equity and justice concerns.

### Procedural debates

Legitimacy can be negotiated through procedural elements of governance, sometimes referred to as ‘input’ and ‘throughput’ components (Scharpf 1999, 2000; Risse 2006; Mees et al. 2014; Schmidt 2013). This is conceptually tied to ‘representational deliberation’ (“Representative deliberation” section) and the inclusiveness and fair representation of different interests. Indeed, skewed representation may result in both procedural and distributive inequities (e.g. Paavola 2008). Thus, the effectiveness and equitability of the rules and procedures shaping the decision-making process is highly relevant.

A requisite for procedural justice, and legitimacy more broadly, is accountability (Lawrence et al. 1997; European Commission 2001; Risse 2006; Birnbaum 2016). In the pursuit of procedural justice, it argued that stakeholders should be equally able to challenge decisions that have been made, exercise their legal rights, and hold designated actors to account (Spagnuolo 2011; Schmidt 2013; Goytia et al. 2016). Procedural and substantive fairness are embedded principles that denote equal opportunity amongst stakeholders to influence the decision-making process and due consideration of all interests in the resulting outcome (Paavola and Adger 2006; van Buuren et al. 2014). These principles are also enshrined in legal documents (e.g. Aarhus Convention 1998). Nonetheless, securing accountability may be particularly challenging in the context of complex environmental problems characterised by spatio-temporal interdependencies, cross-scale interactions, and high uncertainty (Biermann and Gupta 2011; Spagnuolo 2011; Cosens 2013), as well as across private-public partnerships (Dellas 2011).

Beyond the judiciary process and traditional pathways for seeking democratic accountability, socio-political mechanisms provide alternative pathways for ensuring accountability and appear to be coming more common place, such as independent public inquiries, ‘media trials’, and citizen juries (van Kersbergen and van Waarden 2004; Klijn and Skelcher 2007; Baber and Bartlett 2009; Sørensen 2010; Hahn 2011). With these blurring boundaries of accountability, Birnbaum (2016) distinguish traditional hierarchical accountability (e.g. electoral accountability) from emerging forms of participatory accountability grounded in citizen participation. However, accountability must also be pursued within the context of procedural justice. Indeed, the potential drawback of a growing culture of scrutiny is the risk of unfair attributions of blame (Butler and Pidgeon 2011; Smith et al. 2017; Alexander et al. 2016b).

In order to determine attribution, transparency is an important precondition (Mason 2008; Hahn 2011; Mees et al. 2017) and fundamental to procedural-based legitimacy (Esty 2006). However, certain authors have called for more nuanced attention to transparency (Gupta 2010; Mitchell 2011), arguing that ‘different governance ends require differently designed transparency policies, with varying implications for whether and how accountability and legitimacy can be secured’ (Biermann and Gupta, 2011: 1858). In an attempt to clarify the legal obscurity around the principle of transparency, Buijze (2013) discern three common themes related to the availability, accessibility, and comprehensibility of information. Therefore, access to information can be thought of as an additional prerequisite (Fig. 1). Looking to flood risk governance, there are examples where both transparency and access to information are aligned to legitimacy concerns. For example, speaking about the French CAT-NAT regime for insurance, Suykens et al. (2016) comment that the lack of transparency in declarations of natural disasters undermines the system’s legitimacy. Transparency is seen as an essential pathway for promoting democracy (i.e. informed deliberation, accountability, and protection of individual rights), increasing trust and legitimacy, as well as improving the quality of decisions and facilitating acceptance (discussed further in “Legitimacy as a measure of socio-political acceptability” section).

### Distributive debates

Social equity is predominantly discussed in terms of ‘winners and losers’ and related to the spatio-temporal distribution of outcomes (e.g. Adger et al. 2005), as well as in the context of burden sharing and distributive justice (Table 1). In terms of FRM, this is associated with the distribution of (i) flood risk, (ii) financing FRM, (iii) recovery mechanisms, and (iv) responsibilities (Penning-Rowsell and Priest 2015). How these are addressed across different countries (e.g. with varying exposures to risks, cultural attitudes, political ideologies, and administrative structures) is notably varied and manifest in different burden sharing arrangements and legal principles (e.g. Termeer et al. 2011).

For instance, in the Netherlands, flood protection is a constitutional right and enacted through legal safety standards (e.g. 1 in 10,000 recurrence interval along the coast), with variations depending on the type of risk and cost-benefit appraisals (Van Rijswick and Havekes 2012). These somewhat utilitarian concerns are firmly situated within an institutionalised egalitarian stance on national safety and compulsory national solidarity against flooding (Van Alphen 2015; Keessen et al. 2016). However, national solidarity does not guarantee equal benefits to all. Drawing attention to the undebated and silent discourse of social justice in the Dutch context, Kaufmann et al. (2016) highlight disparities between citizens residing inside and outside protected areas, property and land owners, and nature conservation organisations. Despite observing numerous principles of justice and variations between types of floods (e.g. fluvial, coastal, and surface water), this is rarely made explicit in Dutch FRM. In light of projected increases of flooding in response to climate change, disparities are likely to increase; thus, there is a need to bring discussions of justice and debates on burden sharing to the fore (Kaufmann et al. 2016). Similarly, Keessen et al. (2016) argue for transparent public debate concerning the normative and moral foundations of solidarity in discussions of adaptation measures and fair funding arrangements in the Netherlands.

In contrast to the Dutch system, the absence of statutory rights to flood protection and mandated safety standards in England means decisions about the distribution of resources for flood defences are primarily derived from cost-benefit analysis. Whereas, historically, this was guided by efforts to maximise economic efficiency and utilitarianism (Johnson et al. 2007), with the introduction of Partnership Funding in 2012, this has now been complemented by egalitarian principles of justice and efforts to provide equal opportunity in the distribution of resources. In theory, this can be considered to be a fairer approach from the perspective of those at-risk; however, the extent to which communities truly have equal opportunities to funding has been called into question, particularly as the ability to mobilise social capital and resources to secure funding at the local scale may vary from place to place (Alexander et al. 2016b). To help mitigate these effects, the funding calculator incorporates a deprivation bias to support communities ‘least likely to be able to contribute towards the cost of a flood defence scheme and less able to recover after a flood without additional support from the state’ (pers comms, with former national-level policymaker). This reflects a more Rawlsian perspective on social justice, whereby inequalities are justified for the benefit of those least advantaged (Rawls 2001). Moving beyond the perspective of at-risk citizens, the emphasis on contributions from beneficiaries arguably also instils a fairer approach from the perspective of the tax payer (Thaler and Priest 2014). However, recent research conducted by Adger et al. (2017) shows how perceptions of fairness are contextually varied, particularly when confronted with moral intuitions. Drawing from moral foundations theory, these authors demonstrate the presence of vulnerability-based moral intuitions in England following the winter 2013/2014 floods. In this instance, solidarity was evident through widespread examples of public involvement in recovery efforts and general support for the distribution of additional funding to protect flood-vulnerable communities.

Debates on equity and justice are also relevant for the study of recovery mechanisms from natural hazards, whether provided through private market-based insurance, stated-implemented insurance, or compensation schemes, and can manifest in many ways across countries (see Priest, 2014; Penning-Rowsell and Priest, 2015). Adopting a legal perspective, van Doorn-Hoekveld et al. (2016) examine the influence of ‘preflood’ compensation, i.e. measures used to prevent floods, as opposed to recovery mechanisms (e.g. flood storage areas), upon distributive effects of floods in selected European countries. For example, in the Netherlands and Belgium, expropriation is obliged to compensation for the loss of property value, yet elsewhere, such costs are transferred to the injured party (e.g. England). In turn, it is argued that more equitable management of such distributive costs increases the legitimacy of flood risk governance (van Doorn-Hoekveld et al. 2016). Critical questions have also been asked about the distributive fairness connected to upstream and downstream mitigation or rural-urban divides (Thaler and Hartmann 2016). According to Cosens (2013), conscious recognition of these cross-scale linkages is essential for securing legitimate governance.

As with procedural debates, transparency forms an important precondition for securing distributive justice and enhancing the legitimacy of governance. Inevitably, certain justice principles may be valued and prioritised over others depending on the context of decision-making; however, as stressed by Termeer et al. (2011: 175) ‘to improve the legitimacy of adaptation measures it is important that all stakeholders are informed and can understand the more fundamental choices that have been made before practical measures are undertaken’.

### Embedding equity, justice, and moral reasoning in flood risk governance

Moving forward, we propose several signposts to promote critical reflexivity and explicate equity, justice and moral reasoning underlying flood risk governance. Also highlighted is the importance of understanding how these may shape public perceptions of legitimacy and in turn inform strategies for mitigating perceived legitimacy deficits.

- What is the nature of underlying principles of justice in certain aspects of FRM?
- How are resources for FRM allocated and justified?
- How are responsibilities distributed in terms of risk management? Recovery from floods? Are the distribution of responsibilities regarded to be fair across stakeholders?
- To what extent do different groups have equal access to procedural justice?
- To what extent are inclusion/exclusion criteria for participatory governance justified in relation to equity, justice, and moral debates?
- How do perceptions of fairness, justice, and moral ‘right and wrongs’ vary across groups? Are there procedures in place to assess and monitor societal perceptions of these?
- How are perceptions of injustices and moral ‘wrongs’ managed?
- Are checks and balances in place to ensure the fair attribution of accountability?

## Legitimacy as a measure of socio-political acceptability

Throughout this analysis, we observed the recurring framing of legitimacy in terms of acceptance, or sometimes expressed as output legitimacy (Scharpf 2000; Sørensen and Torfing 2005; Adger et al. 2005; Bernstein 2005; Lindgren and Persson 2010; Biermann and Gupta 2011; Schmidt 2013). When deconstructed, we revealed a number of different underlying factors shaping the socio-political acceptability of governance, broadly grouped as follows:

- Governability (a measure of capabilities)—related to goal attainment, efficiency, problem-solving capacity, capacities for learning, as well as normative and cultural expectations
- Authority and the distribution of responsibilities

Governability refers to the performance capacity of the governance network, or more importantly, perceptions of performance capacity, which in turn influence its legitimation. A range of criteria may dictate how performance is judged, whether in terms of problem-solving capabilities, goal attainment, or efficiency (e.g. Risse 2006; Biermann and Gupta 2011; Mees et al. 2017), as well as perceptions of fairness (“Equity and justice in the construction of legitimacy” section). More recently, these views have broadened to take into account the ability to self-reflect, innovate, learn, and implement change, which are widely regarded as essential for cultivating adaptive capacities (e.g. Voß and Bornemann 2011; Fournier et al. 2016). According to van Kersbergen and van Waarden (2004), whereas input legitimacy is dependent on effective accountability, this must be balanced against governability, i.e. the capacity to deliver socially valued outcomes by addressing the problem at hand.

Stakeholder participation is widely credited with the latter and seen as an essential pathway for increasing the quality of governance decisions and resulting output by drawing from multiple types of knowledge (Scharpf 2000). An example in FRM is when public participation is used to inform specific measures of defence or mitigation. In England, Alexander et al. (2016b) document how public exhibitions have been used to demonstrate flood modelling and facilitate a dialogue between the public and risk management authorities in the Hull and Haltemprice catchment, with some instances where local knowledge has help validate flood models and inform the location of defence works. In this sense, participation is employed as strategy for legitimising pre-determined actions (also see Few 2007; Birnbaum 2016). On the flip side to this argument, participation may also pose a potential threat to output legitimacy if it results in inefficiencies and the inability to act (Höreth 2001; Risse 2006; Lindgren and Persson 2010; Dellas 2011). A good example of this is the case of the IJsseldelta in the Netherlands and the formation of an adaptation strategy, studied by van Buuren et al. (2014). Here, the authors report how participatory interaction and principle of social learning can cast doubt on the credibility of proposed measures and undermine output legitimacy.

Legitimacy may also be judged on the basis of normative and cultural expectations. Bernstein (2011: 19) draw attention to the sociological construction of legitimacy from the lens of political economy, emphasising that ‘what constitutes legitimacy results from an interaction of the community of actors affected by the regulatory institution, i.e. the public who grant legitimacy, with broader institutionalized norms – or social structure – that prevail in the relevant issue area’. Cashmore and Wejs (2014) introduce the notion of normative legitimacy in the context of climate change planning and its moral construction through the perceived social obligations of institutions. Expectations are thus ‘founded upon a belief in the appropriateness of certain social norms’ (e.g. protection of vulnerable people) (Cashmore and Wejs 2014: 3). Flood risk management is also attached to perceived social contracts between the State and its citizens, and most notably, the expectation that the state should protect the population. A ‘breach’ in this contract, bought about by the occurrence of flooding, can often spark discourses on what is deemed to be socially (un)acceptable. Smith et al. (2017) demonstrate this in the context of the winter floods on the Somerset Levels in the UK, which prompted major discord between the local community and governing authorities, and led to calls for policy reversals with regard to dredging. This example highlights an additional challenge when social expectations are at odds with legal obligations and policy trajectories. In this context, mitigating the ‘legitimacy gap’ arguably needs to become a process of negotiation and expectation management.

Cashmore and Wejs (2014) also define an additional type of legitimacy from the field of psychology, referred to as cultural-cognitive legitimacy, which can also be interpreted within the umbrella of acceptance. Here, legitimacy is not challenged unless there is a change in the routine and *culturally accepted* and expected way of doing things. When applied to the study of climate change planning in Aarhus, Denmark, normative legitimacy appeared to be less salient than cultural-cognitive legitimacy, with evidence suggesting that climate change planning is legitimised (amongst the business community and political elite) through alignment to existing structures and discourses of economic and ‘green’ growth. However, as demonstrated by van Buuren et al. (2014), aligning FRM with other agendas can also ignite controversies and resistance to adaptation schemes amongst other types of stakeholders (in this case rural communities).

Our analysis also revealed how acceptance can be formulated in terms of authority and acceptance of the governance arrangement as an authoritative voice and also influenced by the governability factors aforementioned (Bernstein 2005; Adger et al. 2005; Lockwood et al. 2010; Lindgren and Persson 2010; Biermann and Gupta 2011; Eriksen et al. 2015). For example, Mees et al. (2014) define legitimacy ‘as the acceptance of authority and justification of political power’ (p. 672). The legitimation of governance is partially influenced by its representativeness. As asserted by Klijn and Skelcher (2007), the representativeness of participants is integral if they (and the governance arena more broadly) are to gain legitimacy and acceptance as ‘legitimate players’, both amongst the constituency affected and within the political system. Authority is also steered through interactions and ‘legitimized, reinforced and challenged through the use of knowledge’ (Eriksen et al. 2015: 529). Therefore, stakeholder participation can play an important role (Paavola 2008).

In addition, acceptance of authority may also be steered through assessments of output. On these matters, considerable attention has been given the issue of European democracy and democratic legitimacy of the European Union (EU), with discussions centred on its political or democratic legitimacy (in terms of authority), as well as output legitimacy in terms of the capacity to deliver effective solutions to salient issues affecting Member States (Höreth 2001; Lindgren and Persson 2010; Schmidt 2013). According to Scharpf (2000), dissatisfaction with the latter may have in turn undermined the former and account for the perceived loss of democratic legitimacy.

Alongside the acceptance of authority, we also included acceptance of responsibilities (and power) through which authority is gained. Here, we observed evidence to suggest that participatory processes are being employed to facilitate the transfer and acceptance of new responsibilities in environmental management (e.g. Birnbaum 2016). In flood risk governance in particular, local flood risk action is increasingly seen as more important in holistic and sustainable risk-based approaches. Looking across several EU countries, Mees et al. (2016a) document how public participation is employed as part of these efforts to coproduce flood risk governance and disperse responsibilities, particularly amongst direct beneficiaries of FRM.

Societal acceptance of responsibilities and outputs of governance may be influenced by multiple factors. For instance, Mees et al. (2014) show how the output legitimacy of flood adaptation strategies was facilitated by transparent risk and responsibility communication in Hamburg, whereas in Helsinki and Rotterdam, acceptance was attributed to low awareness and underestimations of flood risk. Similarly, Mees et al. (2016b) demonstrate high output legitimacy in Flemish FRM and limited concern with ‘throughput’ participation amongst citizens themselves, largely owing to a prevailing view that FRM is a governmental responsibility. This framing of legitimacy is likely to come into conflict if trends towards public-private risk sharing continue.

Transforming these debates into ‘signposts’ to guide efforts to bridge ‘the legitimacy gap’, this analysis draws attention to the multiple ways in which societal acceptance is influenced. Thus, speaking of legitimacy in terms of acceptability is arguably unhelpful. If challenges are bought against arrangements of governance then a fundamental staring point is to determine the underlying factor(s) shaping this. Dissatisfaction might be attributed to views on participatory quality, perceptions of fairness, moral expectations, or whether the action challenges the politically, socially, culturally accepted ways of doing things. Understanding this is essential for designing strategies to address legitimacy gaps in flood risk governance. Beyond ex post measures, socio-political acceptability could also be proactively encouraged through participatory processes and open dialogue to derive objectives and expectations against which flood risk governance can be reasonably judged.

## Bridging the legitimacy gap

This paper contributes to a growing repository of multi-disciplinary research into the legitimacy of governance, as well as more recent concerns with legitimate flood risk governance in particular (Alexander et al. 2016a; Mees et al. 2017). Recognising the somewhat ambiguous nature of legitimacy and multitude of meaning, we performed a comprehensive and interpretive analysis of the literature from which four core inter-locking themes were discerned in relation to *representative deliberation*, *procedural and distributive debates of social equity and justice*, and *socio*-*political acceptability*. Furthermore, this paper has shown how these themes may emerge through different aspects of flood risk governance and perspectives.

In an effort to bridge the gap between academia and policy and practitioner communities, a number of critical questions (or ‘signposts’) are presented to support the translation of these theoretical discussions into practical governance. Ultimately, what is called for is the practice of reflexive governance, whereby actors are encouraged to scrutinise and make transparent ‘their underlying assumptions, institutional arrangements and practices’ (Hendriks and Grin, 2007: 333). This is particularly warranted in the context of flood risk governance where a broad range of legitimacy dilemmas exist and appear to be in a state of flux, with projected increases in flooding igniting re-evaluations of burden-sharing arrangements across public-private parties (Driessen et al. 2016). Transparent and open reflexivity can assist in the identification, deliberation, and negotiation of such legitimacy dilemmas across involved stakeholders, but may also in turn minimise the potential detriment of legitimacy deficits (particularly where these may undermine resilience goals; e.g. van Buuren et al. 2014).

Although legitimacy can be conceptualised as a multi-faceted problem, we acknowledge that the salience of these may vary depending on the aspect of decision-making, the actors involved, depending on spatio-temporal scales and across different socio-cultural normative settings through which legitimacy is (de)constructed. Further empirical studies are required to elaborate on these further and sustain momentum for legitimacy-based research. Moreover, we wish to encourage more action-based research to assist in the uptake and practice of reflexive flood risk governance, whereby legitimacy is brought to the fore.

## Footnotes

1 For transparency and replicability, the full search string in Scopus was as follows: TITLE-ABS-KEY (legitimacy* AND governance) AND (LIMIT-TO (DOCTYPE, “ar”) OR LIMIT-TO (DOCTYPE, “ip”)) AND (LIMIT-TO (LANGUAGE, “English”)) AND (LIMIT-TO (SRCTYPE, “j”)) AND (EXCLUDE (SUBJAREA, “MEDI”) OR EXCLUDE (SUBJAREA, “COMP”) OR EXCLUDE (SUBJAREA, “ENGI”) OR EXCLUDE (SUBJAREA, “PSYC”) OR EXCLUDE (SUBJAREA, “NURS”) OR EXCLUDE (SUBJAREA, “BIOC”) OR EXCLUDE (SUBJAREA, “CENG”) OR EXCLUDE (SUBJAREA, “HEAL”) OR EXCLUDE (SUBJAREA, “MATH”) OR EXCLUDE (SUBJAREA, “MATE”) OR EXCLUDE (SUBJAREA, “IMMU”) OR EXCLUDE (SUBJAREA, “PHAR”) OR EXCLUDE (SUBJAREA, “PHYS”) OR EXCLUDE (SUBJAREA, “Undefined”)) AND (LIMIT-TO (SUBJAREA, “SOCI”) OR LIMIT-TO (SUBJAREA, “ENVI”)) AND (LIMIT-TO (EXACTKEYWORD, “Legitimacy”)).
